# Growth and health status of *Pangasionodon hypophthalmus* reared under manipulated photoperiod conditions

**DOI:** 10.12688/f1000research.28259.2

**Published:** 2021-05-14

**Authors:** Windarti Windarti, Bintal Amin, Asmika H. Simarmata

**Affiliations:** 1 Fisheries and Marine Science Faculty, Universitas Riau, Pekanbaru, Riau, Indonesia

**Keywords:** light, hematology, tissue structure, effective fish culture, aquaculture

## Abstract

**Background: **In general, the length of photoperiod affects the physiology of *Pangasionodon hypophthalmus*. This study aimed to understand the growth and health status of this fish reared under manipulated photoperiods.

**Methods:** The study was conducted between June to August 2020[A1] . Three treatments were applied: control (natural photoperiod); 18 hours of darkness (18D6L; tanks were placed under a dark tarp tent for 18 hours); and 24 hours of darkness (24D0L; tanks were placed under a dark tarp tent continuously). Three replications were performed per treatment. At baseline, fish were approx. 7.5 cm  [A2] total length (TL) and 4 g body weight (BW), and were reared in circular plastic tanks (100 L; 30 fish/tank) with aerators and filters, and fed with commercial fish feed pellet (2 times/day to satiation). Fish growth and survival were studied once/week, and blood and tissue samples were taken at the end of the experiment (8 ^th^ week). Tissue was formalin fixed and HE stained.

**Results:** The survival of fish in all treatment was 100%. The fish reared in 24D0L and 18D6L grew better than control, achieving mean TL of 23 cm and BW of 98 g (control = 19 cm TL and 72 g BW). There was no difference in hematology condition or tissue structure between the three groups. Tissue structure of gill, kidney and liver were normal, but light abnormality due to parasites was present in the gill of fish reared in 24D0L. Blood samples for all three groups showed mean red blood cell count of 1,800,000 cells/ml and white blood cell (WBC) count of 55,200 cells/ml. WBC consisted of lymphocyte 65%, monocyte 24%, thrombocyte 6%, neutrophil 3%, eosinophil 1% and basophile 1%.

**Conclusion:** Data obtained indicate that a short photoperiod improves the growth of *P. hypophthalmus* fish and does not negatively affects their health.

## Introduction

In Riau, Indonesia, *Pangasionodon hypophthalmus* or “ *patin*” fish is one of the most favored fish. This fish is commonly smoked or freshly sold and is a main ingredient for many types of Riau’s traditional cuisine. The fish commonly sold as smoked fish, around 8 to 10 USD/kg or fresh fish that priced around 2 USD/ kg. Due to high demand and high economic value, the fish is commonly cultured ^1^. 

To increase the effectiveness of fish culture, various techniques are commonly applied. For example, fish farmers in Riau feed the fish abundantly or to satiation. Using this technique, the fish may grow well, but the cost of fish feed becomes high. As the fish are provided with a high quantity of food, the amount of uneaten feed and metabolism discharge, such as urine and fish feces, that enter the water may also increase, contaminating water in the surrounding area. Another technique to improve fish culture is the use of chemicals to maintain fish health and boost growth. However, the remains of the chemicals may be present in fish meat and may also pollute the environment.

An environmentally friendly method that can be applied to fish culture is the manipulation of the photoperiod. This technique may be categorized as a “ *natural method*”. The application of this method is cheap, no veterinary medicines used, and it is environmentally friendly. Several studies have proved that this method is effective to improve the growth of several fish species, such as the nocturnal fish *Clarias batrachus* ^2^, *Ompok hypophthalmus* ^3^ and *P. hypophthalmus* reared in tarp pond ^4^. A short photoperiod positively affects the growth of these fish. Fish that are reared under dark condition are shown to be less active in swimming, but react positively toward feed provided ^5, 6^.

Even though dark condition improves the growth of fish, water quality in the rearing tank may poor. As there is no light, phytoplankton are not able to thrive and as a consequence there is no organism consuming organic materials present in the water. High content of organic materials in the rearing media may decrease the water quality and provide suitable conditions for growth of parasites and pathogens of fish. The fish may become more vulnerable towards parasite and pathogen, affecting the health of the fish. Pathogens and parasites will negatively affect the physiology of fish and this may be reflected in the hematology and tissue structure of the fish. According to Fazio ^7^, blood cell count represents a important and powerful tool to diagnose the health status of fish. Hematological condition may be used to evaluate fish health and welfare in cultured fish and in free-living fish ^8^.

Health status, especially blood condition and tissue structure of fish’s main organ such as gill, liver and kidney, has never been reported in the context of photoperiods. In order to understand the effects of shortened photoperiod on *P. hypopthalmus* growth and health status, this study was conducted. Information obtained in this study might be used as a basis to design healthy, effective, and environmentally friendly *P. hypopthalmus* culture conditions.

## Methods

### Study design and fish

This study was conducted at the Aquatic Biology Laboratory, Fisheries and Marine Science Faculty, Riau University, from June to August 2020. The experiments were carried out within the ethical guidelines provided by the research institution and national or international regulations.

Fingerlings of *P. hypopthalmus* were obtained from the hatchery of the Riau Province’s Marine and Fisheries Department in Tibun, Pekanbaru. The fish samples were selected based on their performance. The fish that showed active swimming, no wounds or external parasites and were approximately 7.5 cm total length (TL) and 4 g (BW) were chosen for the research. The density of fish was 30 fish/tank (total of 270 fish in 9 tanks = 3 per treatment).

Fish were reared in 110 L circular grey plastic tanks that were filled with 100 L freshwater obtained from a bored well. The tanks were fitted with an aerator, recirculating pump and filter. During the research, the fish were feed with commercial fish feed pellet (F999 with 35% protein content for weeks 1–5; and F718-1 for weeks 6–8; PT Central Proteina Prima Tbk Indonesia). The feed was given two times/day, in the morning (06.00 am) and in the afternoon (06.00 pm) or until, *ad libitum* until satiation. The feeding was stopped when most of fish were not positively responses to feed provided. In feeding the fish, for each experimental tank, a plastic bottle was fill with 100 grams of feed pellets and it is used to feed the fish. If the feed is finished before a week period, the bottle was again fill with 100 grams of pellets. By the end of the week the feed remains in the bottle were weighed. The amount of feed eaten by the fish in each treatment/ week was calculated using this formula:

The feed eaten/ week = total weight of feed pellet put in the bottle – the weight of feed pellet remains by the end of the week.

To maintain the water quality, any feed remains were removed every day. The filter was washed or replaced once/week, and around 25% the water volume was removed and replaced with clean freshwater once/week.

### Treatments

Three treatments (each with three replications) were applied: 24D0L, fish were cultured under continuous dark condition (24 hours of darkness); 18D6L, fish were cultured with 18 hours of darkness and 6 hours of light; control (Co), the fish was reared under natural photoperiod. The rearing tanks were grouped based on the photoperiod treatments and in each group the tanks were placed randomly based on lottery method.

The light used in this study was natural sunlight, while the dark condition was created by placing the tanks under dark blue colored tarp tent. In 24D0L treatment, the tanks were placed under the tent continuously, which was only opened during feeding times (around 5 minutes/feeding time), two times/day in the morning and in the evening. In 18D6L treatment, the tanks were exposed to natural light for 6 hours (7 am to 1 pm). For the natural photoperiod treatment, the tanks were exposed to sunlight and were placed under transparent plastic tent to prevent evaporation and to avoid rainwater input.

### Data collection and analysis

Fish growth (TL and BW) was measured at baseline and then monitored weekly. Each week, 3 fish from each tank were randomly sampled. The total length was measured using a USA model plastic ruler with 1 mm accuracy and the body weight was measured using a digital scale (0.01 gr). The growth of fish, specific growth rate (SGR) and Feed Conversion Ratio were calculated using these following formulas:

$$\text{Mean}\;\text{Weight}\;\text{Gain}\;(\text{MWG})=\text{Final}\;\text{Mean}\;\text{Weight}\;({\text{W}}_{1})-\text{Initial}\;\text{Mean}\;\text{Weight}\;({\text{W}}_{0})$$

$$\text{Mean}\;\text{Length}\;\text{Gain}\;(\text{MWG})=\text{Final}\;\text{Mean}\;\text{Length}\;({\text{L}}_{1})-\text{Initial}\;\text{Mean}\;\text{Length}\;({\text{L}}_{0})$$

$$\text{Specific}\;\text{Growth}\;\text{Rate}\;\text{(SGR)}\;=\frac{\text{(Ln}\;\text{Final}\;\text{Mean}\;\text{Weight}-\text{Ln}\;\text{Initial}\;\text{Mean}\;\text{Weight})}{\text{Length}\;\text{of}\;\text{rearing}\;\text{trial}\;\text{(days)}}$$

$$\text{Feed}\;\text{Convertion}\;\text{Ratio}\;\text{(FCR)}\;=\frac{\text{Feed}\;\text{consumed}\;\text{by}\;\text{fish}}{\text{Weight}\;\text{gain}\;\text{by}\;\text{fish}}$$

Blood and tissue sampling were conducted two times, at baseline and in the 8 ^th^ week (end of the research period). Three fish from each tank were taken and their blood were obtained; fish were anesthetized using clove oil (5 drops/L). As the fish were sedated (inactive and no response to touching), blood was then taken from the caudal vein, by inserting an EDTA (Merck) 10% moistened syringe. Blood samples was kept in EDTA moistened vials, in a cool box filled with crushed ice. Total erythrocytes and leukocytes were counted using a Neubauer hemocytometer and then were calculated ^9^ and analyzed ^10^. Hematocrit and leucocrit levels were determined using heparinized micro-hematocrit capillaries that was centrifuged at 12,000 rpm for 3 minutes. To calculate the hematocrit or leucocrit levels, the length of the column of packed red cells or the packed white blood cells was measured and divided by the length of the whole column of blood (cells and plasma) and then multiply this number by 100%. Hemoglobin content in blood was measured using Sahli method ^11^.

Tissue samples (gill, kidney and liver) were taken from three different fish/tank at the end of the research period. The samples were processed for histological study ^12^. After tissue removal, they were fixed in 10% formalin for 48 hours and in 4% formalin for three days. Then the samples were processed using alcohol series and paraffin, 5µm sliced and hematoxylin-eosin stained. The histological study of the tissue was conducted using a binocular microscope Olympus CX-21. Any abnormality in the tissue was noted.

Data collected were analyzed with Microsoft Excel (to create graphics) and SPSS (to calculate ANOVA of growth data).

## Results

During the research, survival of the fish was monitored every day. There is no difference in the survival of fish in all treatments applied. Survival was 100%, and no fish showed any behavioral abnormality, i.e. actively swimming and responding well to the food provided.

**Figure 1.**
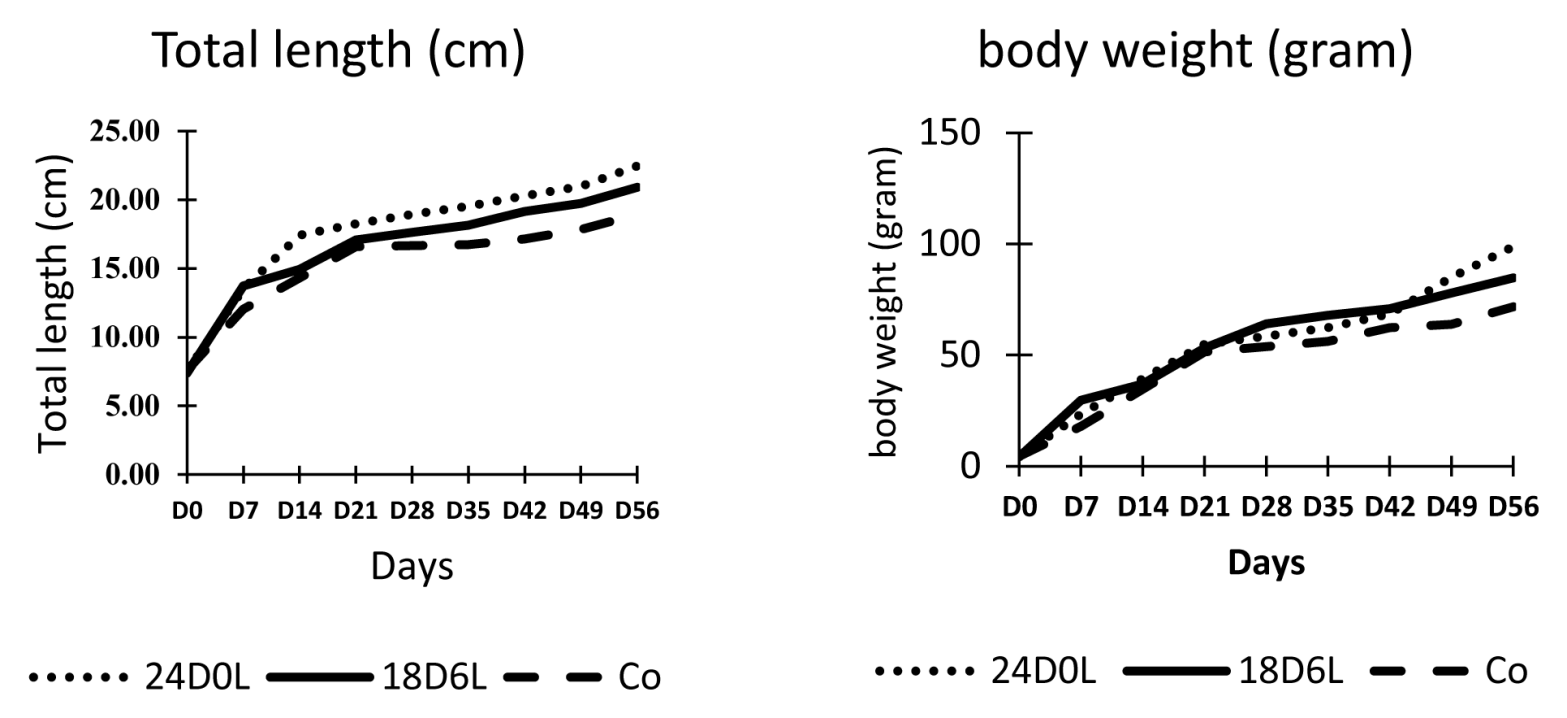
Total length and weight of *Pangasionodon hypophthalmus* reared under manipulated photoperiod. 24D0L, 24 hours of darkness; 18D6L, 18 hours of darkness; Co, control – normal conditions.

Unlike survival, the growth of fish varied according to treatment ( Figure 1). Fish reared in shortened photoperiod (24D0L and 18D6L) exhibited better growth than fish reared under natural photoperiod (control). By the end of the experiment, the fish that were reared under continuous dark (24D0L) were longer and heavier than control fish, but were not that different compared with fish reared in 18D6L. Fish reared in 24D0L and 18D6L treatments were >20 cm TL and 84.74–98.78 g BW, with SGR ranged from 5.39 – 5.52 and FCR 1.51 to 1.62. The growth of control fish was lower, on average 18.93 cm TL and 17.2 g BW with SGR 4.96 and FCR 1.77. Data obtained indicate that the 24D0L treatment resulted in the best treatment condition as the fish has the greatest TL and BW ( Table 1). 

**Table 1.** Total length and body weight of *Pangasionodon hypophthalmus* reared under manipulated photoperiod at the end of the experiment (8 week treatment period).

Treatment	Total length (cm)	Body weight (g)	SGR (%/day)	FCR
24D0L	22.48 ± 1.83 ^ab^	98.78 ± 6.57 ^a^	5.52±0.36	1.51±0.04 ^a^
18D6L	20.91 ± 1.40 ^abc^	84.74 ± 1.55 ^a^	5.39±0.24	1.62±0.04 ^a^
Control	18.93 ± 0.56 ^bc^	71.62 ± 7.64 ^b^	4.96±0.28	1.77±0.09 ^b^

24D0L, 24 hours of darkness; 18D6L, 18 hours of darkness; Control, normal conditions. Mean with standard error followed by different letters are significantly different (P<0.05)

Hematological conditions of the fish in all treatments were similar. There is no difference in the blood condition of all fish treated ( Table 2). The number of red blood cells (RBC) as well as the number of white blood cells (WBC), and hematocrit and leucocrit levels were normal. In all treatments, the number of RBC was around 1,800,000 cells/mL with 25.62–36.63% hematocrit level.

**Table 2.** Hematological analysis of *Pangasionodon hypophthalmus* reared under manipulated photoperiod.

Treatments	Erythrocyte (cells/ mL)	Leucocytes (cells/ mL)	Hematocrit level	Leucocrit level	Hemoglobin (g/dL)
Baseline	1,875,645	55,343	25.62%	1.45%	9
24D0L	1,801,121	55,563	33.79%	1.53%	12
18D6L	1,770,030	54,213	35.16%	1.27%	11
Control	1,781,279	55,657	36.63%	1.54%	10

24D0L, 24 hours of darkness; 18D6L, 18 hours of darkness; Control, normal conditions.

**Figure 2.**
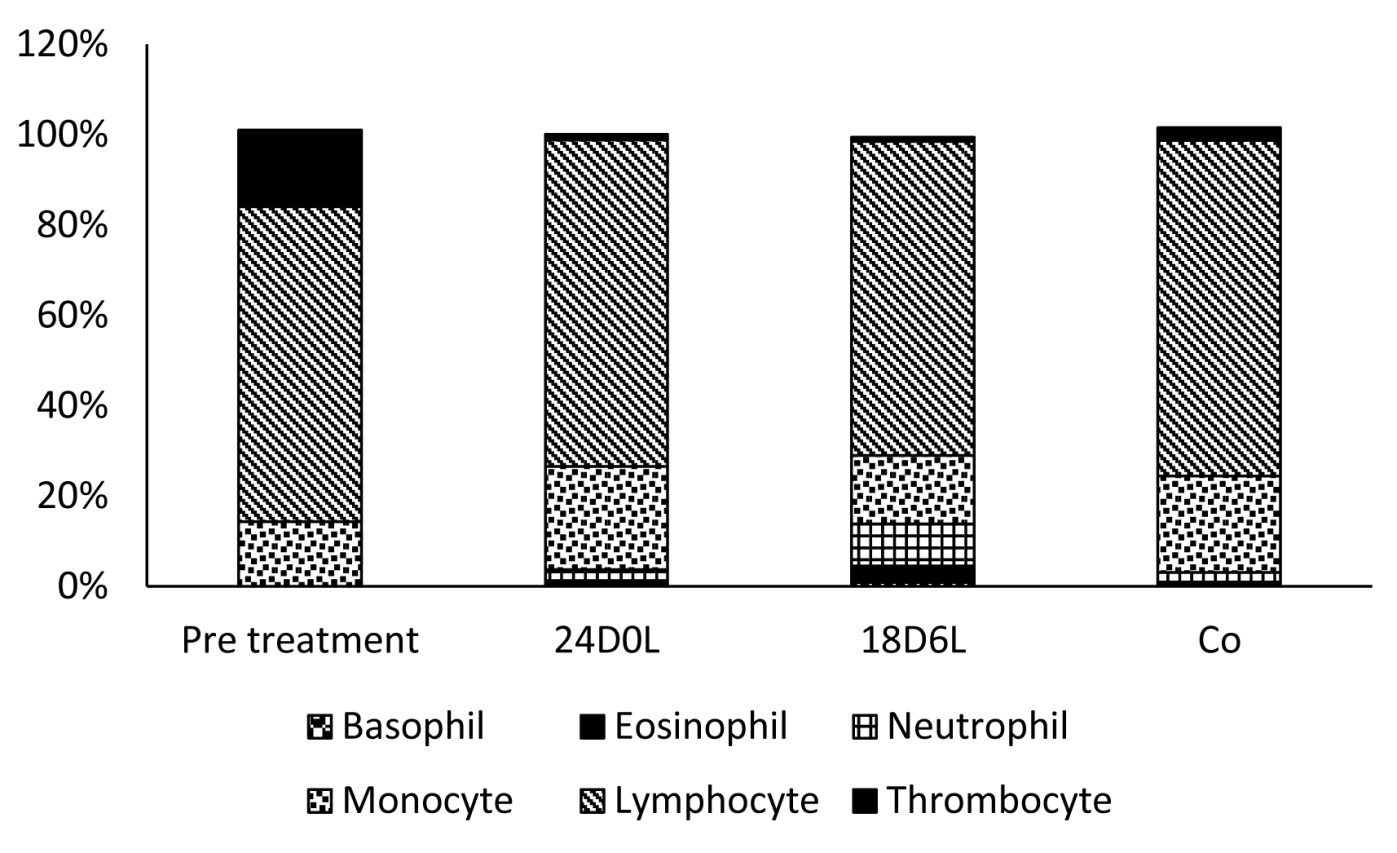
Population of white blood cells types in *Pangasionodon hypophthalmus* reared under manipulated photoperiod. 24D0L, 24 hours of darkness; 18D6L, 18 hours of darkness; Co, control – normal conditions.

Data presented in Table 2 show that the number of WBC and leucocrit level in fish of all treatment are almost the same, ranging from 54,213 to 55,563 cells/ mL and 1.27–1.54%, respectively. WBC cell examination showed that the WBC component of *P. hypopthalmus* consisted of lymphocytes, monocytes, thrombocytes, neutrophils, basophils and eosinophil ( Table 2; Figure 2).

Data presented in Table 3 shows that the most common WBC type present in all treated fish is lymphocytes: lymphocytes, 69.55–74.29%, followed by monocytes (14.31–22.80%), thrombocytes (1.00–17%), and few amount granulated cells (neutrophil, eosinophil and basophil).

**Table 3.** Percentage of white blood cell types in *Pangasionodon hypophthalmus* reared under manipulated photoperiod.

Treatments	Basophil	Eosinophil	Neutrophil	Monocyte	Lymphocyte	Thrombocyte
Baseline	0.01%	0.00%	0.01%	14.31%	69.77%	17.00%
24D0L	0.13%	1.21%	2.43%	22.80%	72.23%	1.33%
18D6L	0.90%	3.66%	9.32%	15.09%	69.55%	1.00%
Control	0.58%	0.38%	2.10%	21.42%	74.29%	2.78%

**Figure 3.**
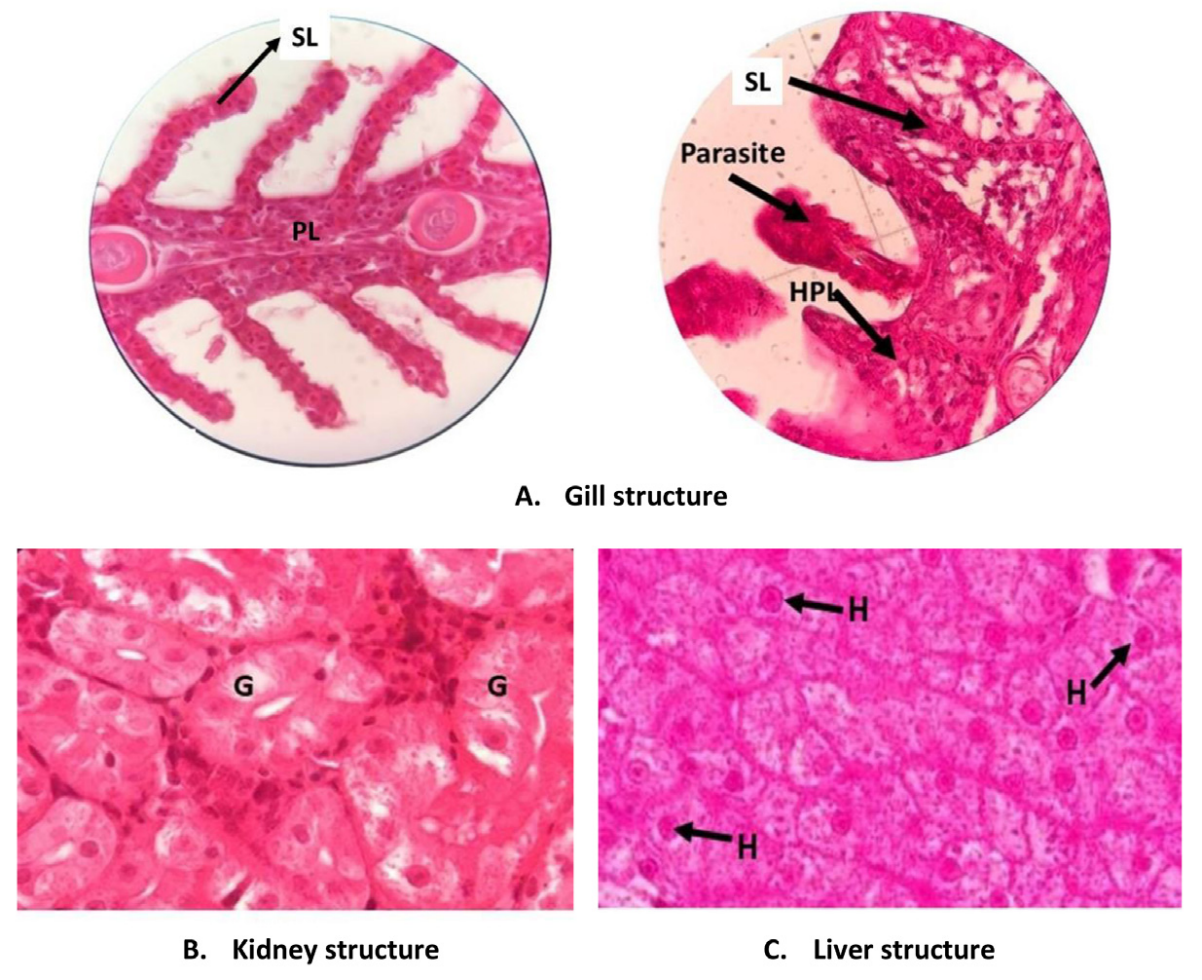
Tissue structure of *P. hypophthalmus* reared under manipulated photoperiod. PL: primary lamellae; SL: secondary lamellae; HPL: hyperplasia; G: glomerulus; H: hepatocyte cells.

Looking at the gill structure of the fish revealed that the fish are healthy ( Figure 3). There is no abnormality such as hyperplasia, hypertrophy, lifting epithelium or hemorrhage. The abnormality in fish gill tissue is mainly caused by environmental factors, including poor water quality, pollutants, and also pathogens and parasites ^13^. Among the 27 fish studied, the only gill abnormality was found in the gills of three fish that that were reared in under 24D0L treatment, in the third replication tank. The gills of these fish are pale with dark red spots due to hemorrhage. Detailed study using a binocular microscope showed that many secondary lamellae exhibited hyperplasia and the half base of the lamellae are fused. Further investigation releveled that there are several parasitic worms, *Dactylogyrus* sp (Monogenea) present in the gills, between the secondary lamellae. *Dactylogyrus* is a parasite that commonly attacks freshwater fish and may cause serious problems in the fish. This worm inserts its sucker to the blood vessel in the gill, causing a wound. The area around the wound becomes damaged as the epithelium proliferates (hyperplasia) and becomes abnormally enlarged (hypertrophy) causing hemorrhage and the cells may be die creating necrosis ^12^. *Dactylogyrus* sp. was presence in one tank only. As each tank has its own circulatory system, the parasite was not distributed to the other research tanks.

The kidney and liver structures of all fish did not show any abnormality. The cells of these organs were normal and there are no deformities such as hemorrhage, necrosis and hypertrophy. These facts indicate that the fish are normal and healthy.

Water quality in each of the tanks was maintained individually. Each tank is completed with a circulatory pump, filter and aerator. The water is maintained by replacing the filter and replacing 25% of the water with clean fresh water. The accumulated materials in the bottom of the tank was cleaned regularly. Through maintenance, the water was of good quality and suitable for the life of *P. hypophthalmus*. As the water was maintained regularly, the manipulated photoperiod treatments applied did not affect the water quality, as well as fish health in general.

## Discussion

In all treatments applied, the survival of the fish was 100%, indicating that the media used in this study and the treatments applied do not provide any negative affect toward the fish. *P. hypophthalmus* is a nocturnal fish that is active at night. In nature, it hides during the day and active in hunting prey at night. Nocturnal fish such as *Ompok hypophthalmus* ^5^ and *P. hypophthalmus* ^6, 13^ are less active in swimming but very responsive to feed provided during darkness. As the fish in this study were used to being active during the dark, the application of shorten photoperiod does not disturb their biological time keeping and as a consequence, they adapt well to the research tank.

The fish that were reared in continuous dark and 18 hours dark grew better than that of fish reared under natural photoperiod (control). The length and weight of fish reared shortened photoperiod were not different, they were higher than that of the fish reared under natural photoperiod. The specific growth rate, however, did not show any difference, while the FCR of fish reared in 24 hours as well as 18 hours dark, shown better performance than that of the control. *P. hypophthalmus* as a nocturnal fish prefers to inhabit dark colored water such as peat water, and therefore continuous dark might be a suitable environment for this fish. Studies on *P. hypophthalmus* behavior indicate that during darkness, fish are less active in swimming but they are very responsive to feed provided ^2, 4^. As the movement of the fish is lower in darkness, the fish may allocate their energy more for growing and as a result they grow faster ^4^. 

In our hematological study, blood parameters of fish in all treatments were categorized as normal, normal number of RBC in fish ranges from 1,000,000 to 3,000,000 cells/ml ^14^. A normal hematocrit level in Teleostei fish is around 30% ^15^. The number of erythrocyte of healthy *P. hypophthalmus* is 1.91–2.83×10 ^6^ cells/mm ^3^ ^16^. As the number of RBCs of an organism determines the dissolved oxygen carrying capacity ^17^, the number of RBCs, hematocrit level and hemoglobin amount of fish in this study indicates that the fish are healthy. Data obtained in this research shows that the application of manipulated photoperiod do not cause any negative effects on fish health in general. The physiological condition, such as RBC values, may be associated with quick movement and high activity of the fish, or is affected by environmental factors such as water salinity ^18^. Hematology parameters of fish may also be used to monitor the fish’s health status in response toward changes in nutrition, disease, water quality and therapy response ^7^. In our recent, the fish were reared in tanks with comparable conditions and the same feed. The only difference in the rearing method was the manipulated photoperiod applied. As there is no difference in the number of RBCs and hematocrit levels between groups, the data obtained in this study demonstrate that the manipulated photoperiod does not affect the blood condition of the fish.

The amount of the WBCs, as well as the leucocrit level can be categorized as normal in this study. In healthy fish, the number of WBCs is <150,000 cells/mL ^11^. The number of WBCs as well as leucocrit level in all treated fish were relatively low, indicating that the fish are healthy and there is no pathogen infection. Even though the WBC number of the fish in this study is slightly higher than that of the cultured *P. hypopthalmus* from Sidoarjo, East Jave, Indonesia (34,600 cells/mL), it is lower than *Edwardsiella tarda* infected fish (77,950) cells/mL ^19^.

In this study, WBCs contained lymphocytes, monocytes, thrombocytes, eosinophils, basophils and neutrophils. The composition of this type of WBC indicate that the fish are healthy. Lymphocytes are the most common leukocytes found in healthy teleosts, and they represent an important function in the cell immunity of fish ^20^. The lymphocyte proportion in healthy cultured fish in Riau is around 70% of total WBC, while the lymphocytes of *Aeromonas hydrophyla* infected fish was around 30% of total WBC ^11^. As the number of phagocytic cell types (monocyte, basophil, eosinophil and neutrophil) in the fish in this present study is relatively low, it means that the fish is not being infected by any pathogen.

Hematological parameters have been used as indicators of fish health ^18^. In our study, hematology data of all fish indicated that the fish were normal and healthy. It suggests that the media and rearing methods applied during the study are suitable for the fish and the photoperiod treatments did not affect the hematology of the fish. 

The health status of the fish in this study was also assessed by examining the tissue structure of the main organs, namely gill, kidney and liver. The condition of these tissue were similar between all treatments. The structure of the organs were normal and did not shown any abnormalities. However, light abnormality, namely hyperplasia was found to occur in the gill of fish reared in continuous dark treatment. The gill’s secondary lamella had suffered from hyperplasia and had become thicker and even fused. This condition may hamper water flow as well as reducing the gill area that is used for oxygen diffusion. As a consequence, the fish may have problem conducting respiration ^21^ and even causing mass death in cultured fish ^22^.

In the gill of fish three fish reared in continuous dark, *Dactylogyrus* (Monogenean) parasites were found ( Figure 3). The abnormalities in the gill of the fish in this study seemed related to the presence of parasites and not related to manipulation of the photoperiod. The presence of the parasite might be related to the general condition of the water in the tank. In the 24D0L treatment, there is almost no light penetrating the water. The lack of light may hamper the growth of phytoplankton that are able to use organic materials in the water to support their life, and which reduce the organic material content in the tank. Due to the absence of phytoplankton, the organic materials in the tank may accumulate and the water condition worsens. The rich organic material water is a suitable habitat for parasites, but this water condition causes stress in fish ^22^. As the fish becomes weak due to stress, the parasite is able to attack the fish and lead the fish to worsen further or even die. The parasitized fish became pale with excess mucus throughout their body, increasing respiration, causing imbalanced swimming, and causing ulcers at the fin edge ^21^. In this study, the parasitized fish became less responsive to feed provided, but none of them died, suggested that the treated fish are able to cope with the parasite. These data indicate that the application of manipulated photoperiod in general improved the growth of the fish, but fish health in general is not negatively affected. 

## Conclusion

Data obtained in this study showed that the application of 24 hours of darkness is effective in improving the growth of *P. hypopthalmus*. The fish reared in the dark grew better than the fish reared under 18 hours dark or a natural photoperiod. The manipulated photoperiod treatment did not have any negative impact on the hematology, tissue structure or general health of the fish. It is recommended to apply the 24 hours dark treatment to culture *P. hypopthalmus*.

## Data availability

### Underlying data

Figshare: Hematology data, https://doi.org/10.6084/m9.figshare.13601417.v1 ^23^.

This project contains the following underlying data:

- Length data at baseline and weekly (n=27);
- Body weight data at baseline and weekly (n=27);
- Hematological analysis and WBC composition at baseline and week 8 (n=27).

Figshare: Tissue structure, https://doi.org/10.6084/m9.figshare.13602104.v1 ^24^.

This project contains the following underlying data:

- Uncropped and unedited tissue structure slide images for gills (n=27), kidney (n=27) and liver (n=27).

Data are available under the terms of the Creative Commons Attribution 4.0 International license (CC-BY 4.0).
