# Supplementary material for: Growth and health status of Pangasionodon hypophthalmus reared under manipulated photoperiod conditions
Source: F1000Res. 2021 May 14;10:154. Originally published 2021 Feb 26. [Version 2] doi: 10.12688/f1000research.28259.2 (PMC8261761; doi:10.12688/f1000research.28259.2)
Supplement: Peer review history

# Peer review: Reviewer response for version 2

**DOI:** 10.5256/f1000research.56748.r87579

**Published:** 2021-07-06

**Authors:** Wan Hee Cheng

**Affiliations:** 1 Faculty of Health and Life Sciences, INTI International University, Nilai, Malaysia

**Abstract**

- Most components are acknowledged in this section.
- Suggest writing the L and D for photoperiod in full when they first appear (e.g., light (L) and dark (D)).

**Introduction**

- No issues in content but suggest checking for grammatical errors.

**Methods**

Study design and fish:

- Any citations or sources of ethical guidelines as mentioned?
- Do indicate what BW is.
- Do check for grammatical errors.

**Results**

- Second paragraph, the phrase “Unlike survival’, what does this mean? Is it a comparison between all the treatments given?
- Do check for grammatical errors.

**Discussion**

- Well discussed but do check for grammatical errors.

Is the work clearly and accurately presented and does it cite the current literature?

Yes

If applicable, is the statistical analysis and its interpretation appropriate?

Yes

Are all the source data underlying the results available to ensure full reproducibility?

Yes

Is the study design appropriate and is the work technically sound?

Yes

Are the conclusions drawn adequately supported by the results?

Yes

Are sufficient details of methods and analysis provided to allow replication by others?

Yes

Reviewer Expertise:

Ecotoxicology

I confirm that I have read this submission and believe that I have an appropriate level of expertise to confirm that it is of an acceptable scientific standard.

# Peer review: Reviewer response for version 2

**DOI:** 10.5256/f1000research.56748.r85401

**Published:** 2021-05-21

**Authors:** Hafrijal Syandri

**Affiliations:** 1 Department of Aquaculture, Faculty of Fisheries and Marine Science, Bung Hatta University, Padang, Indonesia

I accept the Version 1 amendments to this article written by Windarti W, Amin B and Simarmata AH.

Is the work clearly and accurately presented and does it cite the current literature?

Partly

If applicable, is the statistical analysis and its interpretation appropriate?

Yes

Are all the source data underlying the results available to ensure full reproducibility?

Partly

Is the study design appropriate and is the work technically sound?

Partly

Are the conclusions drawn adequately supported by the results?

Yes

Are sufficient details of methods and analysis provided to allow replication by others?

Partly

Reviewer Expertise:

Aquaculture

I confirm that I have read this submission and believe that I have an appropriate level of expertise to confirm that it is of an acceptable scientific standard.

# Peer review: Reviewer response for version 1

**DOI:** 10.5256/f1000research.31255.r80362

**Published:** 2021-03-08

**Authors:** Hafrijal Syandri

**Affiliations:** 1 Department of Aquaculture, Faculty of Fisheries and Marine Science, Bung Hatta University, Padang, Indonesia

**Abstract**: Initial fish length (inches), please convert to units (cm).

**Keyword**: I suggest adding 'Aquaculture'.

**Introduction**: Please add the quantity data about its high demand and economy value of *Pangasionadon hypophthalmus* in Riau Province.

**Methods:**

Study design and fish:

- In the methodology, the research was conducted in June and August 2020, while in abstract was carried out in May and August 2020, please write it correctly.
- The initial length data of fish measured in inches should be converted into cm, because fish growth is measured in units of cm (see. Table 1).
- It is advisable to write down the time for feeding fish in the morning and evening (for example: 09.00 AM and 17.00 PM) and the term adlibitum, please replace it with the feeding rate (% / day).

Data collection and analysis:

- Please write accuracy of ruler i.e. 1 mm to measure the length of the fish and state the type of electronic scale and the model (for example: OHAUS, CT 1200-S USA model).
- Write down the formulas used to analyze growth in total length and body weight, and it is suggested to analyze the specific growth rate (% / day) and feed conversion ratio of fish each treatment.

**Results:**

- Add the results of the analysis of specific growth rate (% / day), and feed conversion ratio (FCR) for each treatment and present them in Table 1.

**Discussion:**

- The growth of *Pangasionadon hypopthalmus* from each experiment has not been discussed, and therefore I recommend discussing it. 

References written in Indonesian are exchanged with English, except for journal names (references numbers 2, 3, 4, 5, 12, 13, 21).

- For example: Windarti BH: Photoperiod manipulation to trigger gonadal maturation of Selais ( *Ompok hypophthalmus*). Research Report, Research Service Universitas Riau, Unpublished, 2017 (In Indonesian), or (In Indonesian with English abstract).

Is the work clearly and accurately presented and does it cite the current literature?

Partly

If applicable, is the statistical analysis and its interpretation appropriate?

Yes

Are all the source data underlying the results available to ensure full reproducibility?

Partly

Is the study design appropriate and is the work technically sound?

Partly

Are the conclusions drawn adequately supported by the results?

Yes

Are sufficient details of methods and analysis provided to allow replication by others?

Partly

Reviewer Expertise:

Aquaculture

I confirm that I have read this submission and believe that I have an appropriate level of expertise to confirm that it is of an acceptable scientific standard, however I have significant reservations, as outlined above.

# Response

**Published:** 2021-04-27

**Authors:** Windarti Windarti

**Affiliations:** Universitas Riau, Indonesia

**Abstract**: Initial fish length (inches), please convert to units (cm).

*I have converted the unit of fish length from inches to cm*

**Keyword**: I suggest adding 'Aquaculture'.

- *I add “aquaculture” into the keywords.*

**Introduction**:

- Please add the quantity data about its high demand and economy value of *Pangasionadon hypophthalmus* in Riau Province.

*I have added information on the quantity data of high demand and economy value of P. pangasionodon*

**Methods:**

Study design and fish:

- In the methodology, the research was conducted in June and August 2020, while in abstract was carried out in May and August 2020, please write it correctly.

*I have revised the research time, from June to August*

- The initial length data of fish measured in inches should be converted into cm, because fish growth is measured in units of cm (see. Table 1).
   
*I have converted the fish length unit, from inch to cm*

- It is advisable to write down the time for feeding fish in the morning and evening (for example: 09.00 AM and 17.00 PM) and the term adlibitum, please replace it with the feeding rate (% / day).

*I have revised the feeding time, at 06.00 in the morning and 06.00 evening. The fish was fed at satiation and I stopped feeding the fish when most of them were not positively response to feed.*

Data collection and analysis:

- Please write accuracy of ruler i.e. 1 mm to measure the length of the fish and state the type of electronic scale and the model (for example: OHAUS, CT 1200-S USA model).

*I used a ruler with 1 mm accuracy, a plastic USA model ruler*

- Write down the formulas used to analyze growth in total length and body weight, and it is suggested to analyze the specific growth rate (% / day) and feed conversion ratio of fish each treatment.

*I have added the formulas used to analyze growth in total length and body weight and also add the information on specific growth rate (% / day) and feed conversion ratio of fish each treatment.*

**Results:**

- Add the results of the analysis of specific growth rate (% / day), and feed conversion ratio (FCR) for each treatment and present them in Table 1.

*I have added the specific growth rate (% / day), and feed conversion ratio (FCR) for each treatment*

**Discussion:**

- The growth of *Pangasionadon hypopthalmus* from each experiment has not been discussed, and therefore I recommend discussing it. 

*I have added the information of fish growth in each treatmen*t

- References written in Indonesian are exchanged with English, except for journal names (references numbers 2, 3, 4, 5, 12, 13, 21).

*I have changed the references written in Indonesian to English*
